# *N*-{4-[(5-Methyl­isoxazol-3-yl)sulfamo­yl]phen­yl}benzamide

**DOI:** 10.1107/S1600536810031132

**Published:** 2010-08-11

**Authors:** Sumera Yasmeen, Shahzad Murtaza, Mehmet Akkurt, Islam Ullah Khan, Shahzad Sharif

**Affiliations:** a Department of Chemistry, University of Gujrat, Gujrat 50700, Pakistan; b Department of Physics, Faculty of Sciences, Erciyes University, 38039 Kayseri, Turkey; c Materials Chemistry Laboratory, Department of Chemistry, Government College University, Lahore 54000, Pakistan

## Abstract

In the title compound, C_17_H_15_N_3_O_4_S, the five-membered isoxazole ring makes dihedral angles of 80.5 (2) and 81.3 (2)° with the two benzene rings, which form a dihedral angle of 39.81 (18)° with each other. A short intra­molecular C—H⋯O contact occurs. The crystal structure is stabilized by inter­molecular N—H⋯O hydrogen bonds, which generate [001] chains, and further consolidated by weak C—H⋯O inter­actions.

## Related literature

The *N*-alkyl­ated moiety is present in many natural products as well as in drugs, see: Wijayanti *et al.* (2010 ▶). For the synthesis and the biological properties of amide derivatives of sulfonamide-type drugs, see: Hussain (2009 ▶). For the crystal structures of similar sulfonamides, see: Shad *et al.* (2008 ▶, 2009 ▶); Chohan *et al.* (2008*a* ▶,*b* ▶); Tahir *et al.* (2008 ▶).
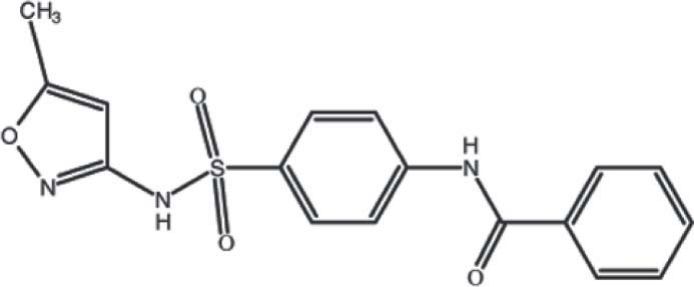


## Experimental

### 

#### Crystal data

C_17_H_15_N_3_O_4_S
*M* *_r_* = 357.39
Monoclinic, 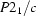

*a* = 8.2407 (6) Å
*b* = 23.5069 (16) Å
*c* = 8.5199 (6) Å
β = 92.155 (3)°
*V* = 1649.3 (2) Å^3^
*Z* = 4
Mo *K*α radiation
μ = 0.22 mm^−1^
*T* = 296 K
0.34 × 0.17 × 0.07 mm

#### Data collection

Bruker Kappa APEXII CCD diffractometer
12756 measured reflections
2865 independent reflections
1544 reflections with *I* > 2σ(*I*)
*R*_int_ = 0.099

#### Refinement

*R*[*F* ^2^ > 2σ(*F* ^2^)] = 0.054
*wR*(*F* ^2^) = 0.135
*S* = 0.99
2865 reflections
227 parameters
H-atom parameters constrained
Δρ_max_ = 0.26 e Å^−3^
Δρ_min_ = −0.33 e Å^−3^

### 

Data collection: *APEX2* (Bruker, 2007 ▶); cell refinement: *SAINT* (Bruker, 2007 ▶); data reduction: *SAINT*; program(s) used to solve structure: *SIR97* (Altomare *et al.*, 1999 ▶); program(s) used to refine structure: *SHELXL97* (Sheldrick, 2008 ▶); molecular graphics: *ORTEP-3* (Farrugia, 1997 ▶); software used to prepare material for publication: *WinGX* (Farrugia, 1999 ▶) and *PLATON* (Spek, 2009 ▶).

## Supplementary Material

Crystal structure: contains datablocks global, I. DOI: 10.1107/S1600536810031132/hb5581sup1.cif

Structure factors: contains datablocks I. DOI: 10.1107/S1600536810031132/hb5581Isup2.hkl

Additional supplementary materials:  crystallographic information; 3D view; checkCIF report

## Figures and Tables

**Table 1:** Hydrogen-bond geometry (Å, °)

*D*—H⋯*A*	*D*—H	H⋯*A*	*D*⋯*A*	*D*—H⋯*A*
N2—H2*N*⋯O4^i^	0.86	2.21	2.788 (4)	124
N3—H3*N*⋯O3^ii^	0.86	2.27	3.075 (4)	156
C7—H7⋯O4	0.93	2.26	2.832 (4)	119
C10—H10⋯O2^iii^	0.93	2.44	3.130 (4)	131

Symmetry codes: (i) 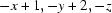; (ii) 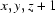; (iii) 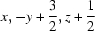.

**Fig. 1.**
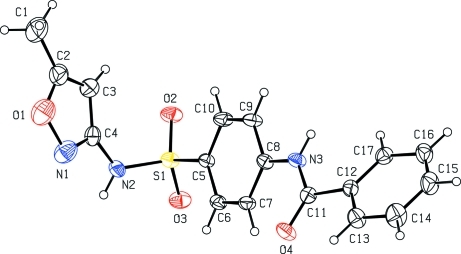
View of (I) with displacement ellipsoids for non-H atoms drawn at the 30% probability level.

**Fig. 2.**
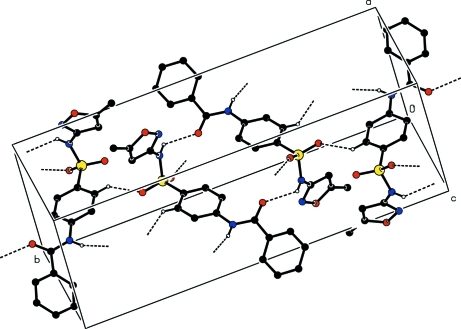
A partial view of the packing of (I), showing the N—H ··· O and C—H ··· O hydrogen-bonding interactions (dashed lines) between the molecules. Hydrogen atoms not involved in hydrogen bonding have been omitted for clarity.

## supplementary crystallographic information

### Comment

*N*-alkylated moiety is present in many natural products as well as in drugs (Wijayanti *et al.*, 2010). Amide derivative of sulfonamide type drugs has been synthesized and biologicaly evaluated (Hussain, 2009). The title compound (I), a new benzamide derivative of sulfamethaxazole, was synthesized by the condensation reaction of sulfamethaxazole with benzoyl chloride.

In (I), (Fig. 1), the bond lengths and bond angles of the molecule are in good agreement with those determined in the similar compounds (Shad *et al.*, 2009; Chohan *et al.*, 2008*a*,*b*; Shad *et al.*, 2008; Tahir *et al.*, 2008). The five-membered isoxazole ring (O1/N1/C2–C4) make dihedral angles of 80.5 (2) and 81.3 (2)° with the two benzene rings (C5–C10) and (C12–C17) which form a dihedral angle of 39.81 (18)° with each other. In (I), The torsion angles C1—C2–C3–C4, O4—C11—C12—C13, C1—N2—S1—C5 and C8—N3—C11—C12 are -177.5 (5), -16.4 (5), -69.4 (3) and 167.0 (3)°, respectively.

The crystal packing of (I) is stabilized by intermolecular N—H···O and C—H···O hydrogen bonding interactions (Table 1, Fig. 2), forming an extended supramolecular network.

### Experimental

Sulfamethaxazole (100 mg, 0.39 mmol) was dissolved in methanol (10 ml) and then benzoyl chloride (45 ml, 0.39 mmol) was added dropwise. The mixture was refluxed for 2 h at temperature ~353 K. The pH of the reaction was maintained at 7–8 by adding pyridine to neuterlize the produced HCl. White precipitates were formed. The precipitates were filtered, washed with plenty of distilled water and crystallized with acetone to yield off-white blocks of (I).

### Refinement

All H atoms bound to N and O atoms were placed in idealized positions and refined using a riding model with C—H = 0.93–0.96 Å and N—H = 0.86 Å, and with *U*_iso_(H) = 1.2 or 1.5*U*_eq_(C,*N*).

### Crystal data

**Table:**

C_17_H_15_N_3_O_4_S	*F*(000) = 744
*M**_r_* = 357.39	*D*_x_ = 1.439 Mg m^−^^3^
Monoclinic, *P*2_1_/*c*	Mo *K*α radiation, λ = 0.71073 Å
Hall symbol: -P 2ybc	Cell parameters from 886 reflections
*a* = 8.2407 (6) Å	θ = 2.5–19.0°
*b* = 23.5069 (16) Å	µ = 0.22 mm^−^^1^
*c* = 8.5199 (6) Å	*T* = 296 K
β = 92.155 (3)°	Block, off-white
*V* = 1649.3 (2) Å^3^	0.34 × 0.17 × 0.07 mm
*Z* = 4	

### Data collection

**Table:**

Bruker Kappa APEXII CCD diffractometer	1544 reflections with *I* > 2σ(*I*)
Radiation source: sealed tube	*R*_int_ = 0.099
graphite	θ_max_ = 25.0°, θ_min_ = 2.5°
phi and ω scans	*h* = −9→9
12756 measured reflections	*k* = −27→27
2865 independent reflections	*l* = −9→10

### Refinement

**Table:**

Refinement on *F*^2^	Primary atom site location: structure-invariant direct methods
Least-squares matrix: full	Secondary atom site location: difference Fourier map
*R*[*F*^2^ > 2σ(*F*^2^)] = 0.054	Hydrogen site location: inferred from neighbouring sites
*wR*(*F*^2^) = 0.135	H-atom parameters constrained
*S* = 0.99	*w* = 1/[σ^2^(*F*_o_^2^) + (0.0555*P*)^2^] where *P* = (*F*_o_^2^ + 2*F*_c_^2^)/3
2865 reflections	(Δ/σ)_max_ < 0.001
227 parameters	Δρ_max_ = 0.26 e Å^−^^3^
0 restraints	Δρ_min_ = −0.33 e Å^−^^3^

### Special details

**Table:**

Geometry. Bond distances, angles *etc*. have been calculated using the rounded fractional coordinates. All su's are estimated from the variances of the (full) variance-covariance matrix. The cell e.s.d.'s are taken into account in the estimation of distances, angles and torsion angles
Refinement. Refinement on *F*^2^ for ALL reflections except those flagged by the user for potential systematic errors. Weighted *R*-factors *wR* and all goodnesses of fit *S* are based on *F*^2^, conventional *R*-factors *R* are based on *F*, with *F* set to zero for negative *F*^2^. The observed criterion of *F*^2^ > σ(*F*^2^) is used only for calculating -*R*-factor-obs *etc*. and is not relevant to the choice of reflections for refinement. *R*-factors based on *F*^2^ are statistically about twice as large as those based on *F*, and *R*-factors based on ALL data will be even larger.

### Fractional atomic coordinates and isotropic or equivalent isotropic displacement parameters (Å^2^)

**Table:**

	*x*	*y*	*z*	*U*_iso_*/*U*_eq_	
S1	0.43740 (12)	0.83329 (4)	−0.22084 (11)	0.0386 (4)	
O1	1.0003 (4)	0.81880 (17)	−0.0861 (4)	0.0844 (17)	
O2	0.4214 (3)	0.77361 (9)	−0.1980 (3)	0.0496 (10)	
O3	0.3429 (3)	0.86108 (10)	−0.3422 (3)	0.0496 (10)	
O4	0.3631 (3)	1.03832 (10)	0.3334 (3)	0.0510 (10)	
N1	0.8855 (5)	0.85441 (16)	−0.1611 (5)	0.0704 (17)	
N2	0.6255 (4)	0.84586 (12)	−0.2644 (3)	0.0437 (11)	
N3	0.3130 (4)	0.94599 (12)	0.3837 (3)	0.0404 (11)	
C1	1.0426 (6)	0.7243 (2)	0.0112 (7)	0.108 (3)	
C2	0.9324 (7)	0.7675 (2)	−0.0637 (5)	0.070 (2)	
C3	0.7813 (6)	0.76770 (19)	−0.1215 (5)	0.0652 (19)	
C4	0.7578 (5)	0.82254 (18)	−0.1798 (4)	0.0452 (16)	
C5	0.4049 (4)	0.86758 (14)	−0.0421 (4)	0.0323 (12)	
C6	0.3831 (4)	0.92538 (14)	−0.0387 (4)	0.0363 (12)	
C7	0.3562 (4)	0.95266 (15)	0.0996 (4)	0.0372 (14)	
C8	0.3494 (4)	0.92116 (15)	0.2385 (4)	0.0344 (12)	
C9	0.3738 (5)	0.86339 (15)	0.2337 (4)	0.0414 (14)	
C10	0.4019 (4)	0.83617 (15)	0.0942 (4)	0.0417 (14)	
C11	0.3098 (4)	1.00198 (16)	0.4204 (4)	0.0364 (12)	
C12	0.2330 (4)	1.01807 (15)	0.5708 (4)	0.0367 (12)	
C13	0.2608 (5)	1.07230 (17)	0.6286 (4)	0.0495 (17)	
C14	0.1852 (6)	1.09083 (19)	0.7601 (5)	0.0651 (19)	
C15	0.0811 (5)	1.0556 (2)	0.8348 (5)	0.0610 (19)	
C16	0.0515 (5)	1.00162 (19)	0.7787 (4)	0.0528 (17)	
C17	0.1291 (4)	0.98271 (16)	0.6476 (4)	0.0430 (14)	
H1A	1.09040	0.73950	0.10680	0.1620*	
H1B	1.12680	0.71480	−0.05920	0.1620*	
H1C	0.98170	0.69070	0.03430	0.1620*	
H2N	0.64370	0.86780	−0.34260	0.0530*	
H3	0.70730	0.73780	−0.12300	0.0780*	
H3N	0.29020	0.92280	0.45780	0.0480*	
H6	0.38660	0.94620	−0.13130	0.0430*	
H7	0.34250	0.99190	0.10140	0.0450*	
H9	0.37120	0.84230	0.32600	0.0500*	
H10	0.41880	0.79710	0.09210	0.0500*	
H13	0.33130	1.09640	0.57800	0.0600*	
H14	0.20470	1.12730	0.79820	0.0780*	
H15	0.03020	1.06810	0.92400	0.0730*	
H16	−0.02050	0.97790	0.82880	0.0630*	
H17	0.11110	0.94590	0.61100	0.0510*	

### Atomic displacement parameters (Å^2^)

**Table:**

	*U*^11^	*U*^22^	*U*^33^	*U*^12^	*U*^13^	*U*^23^
S1	0.0542 (7)	0.0308 (6)	0.0309 (6)	−0.0024 (5)	0.0013 (4)	−0.0030 (5)
O1	0.066 (3)	0.095 (3)	0.091 (3)	0.007 (2)	−0.0124 (19)	−0.007 (2)
O2	0.075 (2)	0.0238 (14)	0.0500 (18)	−0.0059 (13)	0.0019 (14)	−0.0043 (12)
O3	0.068 (2)	0.0470 (17)	0.0330 (16)	0.0020 (13)	−0.0096 (13)	0.0012 (13)
O4	0.078 (2)	0.0357 (16)	0.0409 (17)	−0.0104 (14)	0.0217 (14)	−0.0015 (13)
N1	0.057 (3)	0.063 (3)	0.090 (3)	0.003 (2)	−0.013 (2)	−0.007 (2)
N2	0.051 (2)	0.045 (2)	0.0359 (19)	0.0010 (16)	0.0138 (16)	0.0054 (15)
N3	0.059 (2)	0.0325 (19)	0.0302 (18)	0.0013 (15)	0.0085 (15)	0.0036 (14)
C1	0.067 (4)	0.142 (5)	0.116 (5)	0.039 (4)	0.008 (3)	0.046 (4)
C2	0.066 (4)	0.090 (4)	0.055 (3)	0.021 (3)	0.015 (3)	0.016 (3)
C3	0.049 (3)	0.068 (3)	0.079 (4)	0.009 (2)	0.006 (3)	0.023 (3)
C4	0.045 (3)	0.050 (3)	0.041 (2)	0.003 (2)	0.009 (2)	−0.010 (2)
C5	0.040 (2)	0.029 (2)	0.028 (2)	−0.0013 (17)	0.0020 (17)	0.0018 (17)
C6	0.041 (2)	0.039 (2)	0.029 (2)	−0.0008 (18)	0.0010 (17)	0.0069 (18)
C7	0.051 (3)	0.026 (2)	0.035 (2)	0.0019 (17)	0.0062 (18)	0.0030 (17)
C8	0.041 (2)	0.036 (2)	0.026 (2)	−0.0044 (17)	0.0004 (16)	0.0011 (17)
C9	0.061 (3)	0.029 (2)	0.034 (2)	−0.0002 (18)	0.0012 (19)	0.0085 (18)
C10	0.063 (3)	0.024 (2)	0.038 (2)	0.0000 (19)	0.0026 (19)	−0.0013 (18)
C11	0.037 (2)	0.040 (2)	0.032 (2)	0.0011 (18)	−0.0022 (17)	−0.0021 (19)
C12	0.035 (2)	0.046 (2)	0.029 (2)	0.0032 (18)	0.0004 (17)	0.0007 (18)
C13	0.054 (3)	0.050 (3)	0.045 (3)	−0.008 (2)	0.008 (2)	−0.008 (2)
C14	0.079 (4)	0.066 (3)	0.051 (3)	−0.003 (3)	0.011 (3)	−0.024 (3)
C15	0.066 (3)	0.077 (4)	0.041 (3)	0.008 (3)	0.015 (2)	−0.009 (3)
C16	0.049 (3)	0.071 (3)	0.039 (3)	0.005 (2)	0.008 (2)	0.005 (2)
C17	0.046 (3)	0.043 (2)	0.040 (2)	0.0046 (19)	0.000 (2)	−0.0013 (19)

### Geometric parameters (Å, °)

**Table:**

S1—O2	1.423 (2)	C9—C10	1.377 (5)
S1—O3	1.429 (3)	C11—C12	1.499 (5)
S1—N2	1.634 (3)	C12—C17	1.376 (5)
S1—C5	1.753 (4)	C12—C13	1.383 (5)
O1—N1	1.400 (5)	C13—C14	1.373 (6)
O1—C2	1.346 (6)	C14—C15	1.367 (6)
O4—C11	1.223 (4)	C15—C16	1.375 (6)
N1—C4	1.297 (6)	C16—C17	1.381 (5)
N2—C4	1.396 (5)	C1—H1A	0.9600
N3—C8	1.410 (4)	C1—H1B	0.9600
N3—C11	1.353 (5)	C1—H1C	0.9600
N2—H2N	0.8600	C3—H3	0.9300
N3—H3N	0.8600	C6—H6	0.9300
C1—C2	1.490 (7)	C7—H7	0.9300
C2—C3	1.322 (7)	C9—H9	0.9300
C3—C4	1.392 (6)	C10—H10	0.9300
C5—C10	1.377 (5)	C13—H13	0.9300
C5—C6	1.371 (5)	C14—H14	0.9300
C6—C7	1.367 (5)	C15—H15	0.9300
C7—C8	1.399 (5)	C16—H16	0.9300
C8—C9	1.374 (5)	C17—H17	0.9300
			
O2—S1—O3	119.92 (15)	C11—C12—C13	117.9 (3)
O2—S1—N2	107.63 (15)	C11—C12—C17	123.0 (3)
O2—S1—C5	108.46 (16)	C13—C12—C17	119.0 (3)
O3—S1—N2	104.41 (15)	C12—C13—C14	120.6 (4)
O3—S1—C5	108.76 (16)	C13—C14—C15	120.0 (4)
N2—S1—C5	106.94 (15)	C14—C15—C16	120.3 (4)
N1—O1—C2	108.9 (4)	C15—C16—C17	119.8 (4)
O1—N1—C4	104.0 (3)	C12—C17—C16	120.4 (4)
S1—N2—C4	122.7 (2)	C2—C1—H1A	109.00
C8—N3—C11	127.7 (3)	C2—C1—H1B	110.00
C4—N2—H2N	119.00	C2—C1—H1C	109.00
S1—N2—H2N	119.00	H1A—C1—H1B	109.00
C8—N3—H3N	116.00	H1A—C1—H1C	109.00
C11—N3—H3N	116.00	H1B—C1—H1C	109.00
C1—C2—C3	135.4 (5)	C2—C3—H3	128.00
O1—C2—C3	109.6 (4)	C4—C3—H3	128.00
O1—C2—C1	114.9 (5)	C5—C6—H6	120.00
C2—C3—C4	104.6 (4)	C7—C6—H6	120.00
N1—C4—N2	116.8 (4)	C6—C7—H7	120.00
N1—C4—C3	112.9 (4)	C8—C7—H7	120.00
N2—C4—C3	130.0 (4)	C8—C9—H9	120.00
S1—C5—C6	119.9 (3)	C10—C9—H9	119.00
S1—C5—C10	119.6 (3)	C5—C10—H10	120.00
C6—C5—C10	120.5 (3)	C9—C10—H10	120.00
C5—C6—C7	120.6 (3)	C12—C13—H13	120.00
C6—C7—C8	119.6 (3)	C14—C13—H13	120.00
N3—C8—C7	122.7 (3)	C13—C14—H14	120.00
N3—C8—C9	118.1 (3)	C15—C14—H14	120.00
C7—C8—C9	119.2 (3)	C14—C15—H15	120.00
C8—C9—C10	121.0 (3)	C16—C15—H15	120.00
C5—C10—C9	119.2 (3)	C15—C16—H16	120.00
O4—C11—C12	121.0 (3)	C17—C16—H16	120.00
N3—C11—C12	117.1 (3)	C12—C17—H17	120.00
O4—C11—N3	121.9 (3)	C16—C17—H17	120.00
			
O2—S1—N2—C4	−47.0 (3)	C2—C3—C4—N2	174.6 (4)
O3—S1—N2—C4	−175.5 (3)	S1—C5—C6—C7	179.4 (3)
C5—S1—N2—C4	69.4 (3)	C10—C5—C6—C7	−0.8 (5)
O2—S1—C5—C6	−167.7 (3)	S1—C5—C10—C9	−179.0 (3)
O2—S1—C5—C10	12.5 (3)	C6—C5—C10—C9	1.2 (5)
O3—S1—C5—C6	−35.7 (3)	C5—C6—C7—C8	−0.6 (5)
O3—S1—C5—C10	144.5 (3)	C6—C7—C8—N3	−176.3 (3)
N2—S1—C5—C6	76.5 (3)	C6—C7—C8—C9	1.5 (5)
N2—S1—C5—C10	−103.3 (3)	N3—C8—C9—C10	176.8 (3)
C2—O1—N1—C4	−0.4 (5)	C7—C8—C9—C10	−1.1 (6)
N1—O1—C2—C1	178.2 (4)	C8—C9—C10—C5	−0.2 (6)
N1—O1—C2—C3	0.8 (5)	O4—C11—C12—C13	−16.4 (5)
O1—N1—C4—N2	−175.0 (3)	O4—C11—C12—C17	159.5 (3)
O1—N1—C4—C3	0.0 (5)	N3—C11—C12—C13	165.4 (3)
S1—N2—C4—N1	−143.7 (3)	N3—C11—C12—C17	−18.7 (5)
S1—N2—C4—C3	42.4 (5)	C11—C12—C13—C14	175.7 (4)
C11—N3—C8—C7	−13.4 (6)	C17—C12—C13—C14	−0.4 (6)
C11—N3—C8—C9	168.7 (4)	C11—C12—C17—C16	−174.6 (3)
C8—N3—C11—O4	−11.1 (6)	C13—C12—C17—C16	1.2 (5)
C8—N3—C11—C12	167.0 (3)	C12—C13—C14—C15	−0.2 (7)
O1—C2—C3—C4	−0.7 (5)	C13—C14—C15—C16	−0.1 (7)
C1—C2—C3—C4	−177.5 (5)	C14—C15—C16—C17	0.9 (6)
C2—C3—C4—N1	0.5 (5)	C15—C16—C17—C12	−1.5 (6)

### Hydrogen-bond geometry (Å, °)

**Table:**

*D*—H···*A*	*D*—H	H···*A*	*D*···*A*	*D*—H···*A*
N2—H2N···O4^i^	0.86	2.21	2.788 (4)	124
N3—H3N···O3^ii^	0.86	2.27	3.075 (4)	156
C7—H7···O4	0.93	2.26	2.832 (4)	119
C10—H10···O2^iii^	0.93	2.44	3.130 (4)	131

Symmetry codes: (i) −*x*+1, −*y*+2, −*z*; (ii) *x*, *y*, *z*+1; (iii) *x*, −*y*+3/2, *z*+1/2.
